# The Role of Leadership in Supporting and Mitigating Moral Distress in Nursing: A Scoping Review

**DOI:** 10.1155/jonm/5443770

**Published:** 2025-08-14

**Authors:** Sherrie Bolton, Eileen Willis, Amy-Louise Byrne

**Affiliations:** ^1^School of Nursing, Midwifery and Social Sciences, CQUniversity, Rockhampton, Queensland, Australia; ^2^College of Nursing and Health Sciences, Caring Futures Institute, Flinders University, Adelaide, South Australia, Australia; ^3^School of Nursing, Midwifery and Social Sciences, CQUniversity, Sydney, New South Wales, Australia

**Keywords:** leadership, management, moral distress, nurse leader, registered nurses

## Abstract

**Introduction:** Moral distress is a phenomenon that is increasing in nursing practice. Many factors can contribute to moral distress such as the quality of leadership, the rationing of care, the skill mix assigned to the unit or failure of leadership to respond to the nurse's patient advocacy. While there is a significant amount of research on moral distress in nurses' fewer studies have focused on the role of leadership in managing the moral distress experienced by Registered Nurses.

**Objective:** To explore the relationship between leadership and moral distress in nursing, to identify the role of leadership in supporting and mitigating moral distress.

**Inclusion Criteria:** Studies published from 2022–November 2024 were included. The populations were Registered Nurses, and the phenomenon of interest is the role of leaders in supporting nurses and mitigating moral distress.

**Methods:** A scoping review using Arksey and O'Malley five-stage framework.

**Results:** From the 2892 records retrieved, 20 met the inclusion criteria. Three themes were identified: (1) Qualities of the manager, (2) Workload and resource management, and (3) Culture and values.

**Conclusion:** Nurse leaders play an important role in supporting nursing staff through distressing events and situations. Nurse managers must display qualities of a good leader, including being authentic and ethical in decision-making. Nurse leaders must work between policy and procedure requirements and the needs of individual staff to support a wider culture of safety and advocacy.

## 1. Introduction

The incidence of moral distress is increasing within the profession of nursing [1, 2]. Moral distress occurs when a person knows the right course of action to take but cannot carry this out due to institutional constraints [1]. Moral distress involves compromising a person's moral integrity and core values and as such may lead to poor quality care, attrition from the profession and adverse effects on staff well-being [3, 4].

Many factors contribute to moral distress within healthcare. For nurses, this may occur when the processes put in place impede the care that they believe should be provided to patients [5]. These factors may include the rationing of care, the skill mix assigned to the unit, the quality of leadership and failure of leadership to respond to nurse advocacy for their patients [2, 6].

As such, leadership plays a role in ensuring that moral distress is avoided, mitigated and addressed when it occurs. Novice leaders may not have the experience to make informed nursing decisions, particularly when they are negotiating care, and must work with limited resources [7]. Nurses look to leaders for transparency in decision-making to maintain trust and to ensure adequate care is provided [7]. In addition to managing resources, staffing, clinical care and outcomes, nurse leaders must make space for staff to raise concerns. However, there was a decline in nurse leadership internationally from 2.4% in 2013 to 1.9% in 2017 [1]. In addition, by the year 2030, there will be a shortage of 9 million nurses [2]. The reason for this decline was due to vacancies existing, ethical problems, complexities in nursing being magnified and senior nurse leaders lacking the competency and knowledge to operate in an effective and supportive manner, which also impacts on key performance indicators (KPIs) [1, 2].

Given the unprecedented nature of the COVID-19 global pandemic and its impacts on the health of nursing staff, there have been many recent studies on moral distress. However, beyond COVID-19, moral distress can impact daily upon nursing well-being, and leadership may be one avenue that can support staff through such challenging times. This study seeks to understand the role of the nurse leader in supporting and mitigating moral distress. A scoping review was chosen due to the unknown nature of this research area. There is a large focus on nurse burnout and moral distress regarding the recent pandemic; however, less research exists on the impact of leadership on moral distress and burnout.

## 2. Background

Moral distress is (in part) driven by the requirement of nurses to advocate and escalate care for their patients. Advocacy and autonomy are important parts of the RNs role. Nurses are bound by standards of practice, a code of ethics and a code of conduct, all of which promote working with the patient, escalating concerns as they arise and advocating for the best possible care [8, 9]. These standards and codes assist with guiding nursing practice; however, the structure of the health system, including a lack of staffing and resources, can often mean that nurses and leaders have no recourse when patient advocacy is ignored. Nurses will often look to leaders for backup, support and positive influence when situations such as these arise.

Leadership is identified as a required element in nursing practice [9]. Registered Nurses are leaders and may demonstrate this by escalating their concerns. Effective leadership is defined as where staff feel safe and supported when advocating for their patients and fear and anxiety are alleviated and described as a professional value in the ICN Code of Ethics [10, 11]. Nurse managers (or team leaders and other various names) are also leaders and are often the first point of call for concerns. A leader's actions and attitudes demonstrate their priority for quality and safety improvements for staff and the facility, which in turn may influence attitudes, perception and behaviours of their workforce. Importantly, leadership impacts on staff well-being and is central to creating change and reducing turnover [12]. As such, the need to investigate the role of the nurse leader in supporting staff is clear. Addressing gaps in health service delivery as well as the current and forecasted nursing workforce shortages is of paramount importance. Effective nurse leadership can assist with this current issue, prevent moral distress and retain suitably qualified nursing workforce.

## 3. Methods

### 3.1. Aim and Objectives

This review explored the relationship between leadership and moral distress in nursing to identify how nurse leaders support nurses experiencing moral distress, and how they can mitigate these situations.

The objectives were to:

• Explore the relationship between leadership and moral distress in nursing
• Identify how nurse leaders can support and mitigate moral distress in staff

### 3.2. Review Question

The review question was as follows:

What is the role of nurse leaders in supporting staff through and mitigating moral distress?

### 3.3. Design

This scoping review used the framework of Arksey and O'Malley to explore the literature as the aim was to understand the state of the literature regarding leadership [13]. While there have been scoping reviews on how nurse leaders manage moral distress, there is little research on how they might mitigate or change it in nurse leaders [4, 14]. Arksey and O'Malley define five stages of analysis: identification of the research question; identify relevant studies; study selection; charting the data; and collating, summarizing and reporting the results [13]. To ensure rigour, this review is reported in accordance with the PRISMA Extension for Scoping Reviews (PRISMA-ScR) checklist [15].

### 3.4. Search Methods

A preliminary search of PROSPERO was conducted and no current or commenced systematic reviews on the topic were identified.

A two-step search strategy was used in this review. Firstly, an initial limited search of MEDLINE (Ovid) was undertaken to identify articles on the topic, using a simple PICO framework, demonstrated in Table 1.

In collaboration with a CQUniversity Librarian, the text words contained in the titles and abstracts of relevant articles, and the index terms used to describe the articles were used to develop a full search strategy (see Table A1). The search strategy, including all identified keywords and index terms, was adapted for each database and/or information source.

The databases were searched, including MEDLINE (Ovid), CINAHL (EBSCOhost), Embase (Ovid) and PsycINFO (Ovid). This review only includes papers published in English from 2022 to November 2024, to capture studies post-COVID-19 pandemic and to provide in-depth understanding of leadership and moral distress. All searches were completed on the 24th of October 2024.

### 3.5. Eligibility Criteria and Selection

Both quantitative and qualitative study designs were included. In addition, analytical observational studies including prospective and retrospective cohort studies, case–control studies and analytical cross-sectional studies were considered for inclusion. The review also included descriptive observational study designs including case series, individual case reports and descriptive cross-sectional studies.

**Figure 1:**
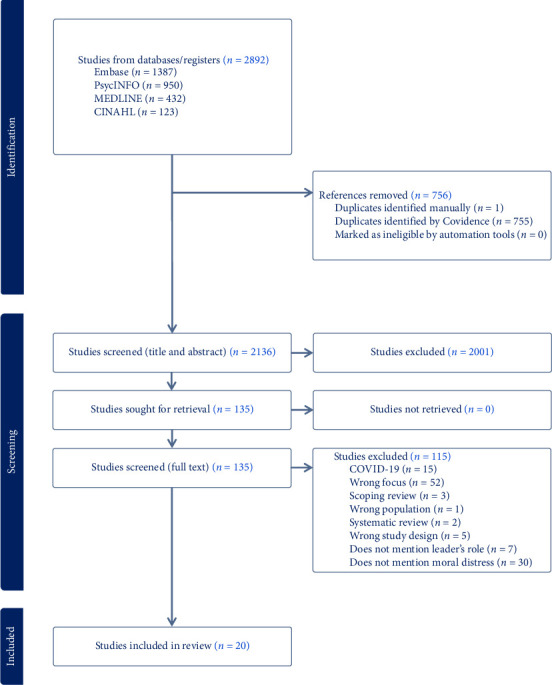
Prisma flow diagram.

Table 2 provides the inclusion and exclusion criteria for the review and considers the participants, concept and context of the research.  Figure 1 displays the Prisma flow diagram.

The full search record for each database was downloaded into Covidence for review and selection. Initial screening, in the form of title and abstract screening, was conducted by all three authors, with each article being independently reviewed by two authors. Conflicts were resolved via consensus. Second round screening, in the form of full text review, was conducted via the same process. Final inclusions were determined via consensus.  Figure 1 demonstrates the search process.

### 3.6. Data Extraction and Analysis

A purpose-built data extraction tool (see Table A2) was created, piloted and used to extract the data. Analysis followed the framework of Arksey and O'Malley whereby initial codes were collapsed into categories, which best captured the total data [13]. Each author independently reviewed the extracted data and created initial codes and categories. Through a consensus meeting, these codes and initial categories were collapsed into final categories. Final categories were determined via a consensus meeting with all authors agreeing on the final version.

## 4. Results

A total of 20 articles are included in this review. The findings are summarized in Table 3, in relation to the role of nurse leader in supporting staff and mitigating moral distress.

### 4.1. Qualities of a Leader

The literature alluded to the need for leaders to have intrinsic qualities which support staff in times of need. Nurse leaders need to possess authenticity, self-awareness and transparency to be effective leader [15]. An authentic leader will adopt a reliable management style, be assertive and aware of their surroundings and collaborate with others to achieve outcomes [15]. A self-aware leader has the capability to recognize their own strengths and weaknesses as well as others, which can result in improved relationships, open disclosure and transparent communication [15].

Flexibility was highlighted as an essential quality of a leader as well as confidence and a high level of nursing knowledge [16, 17]. The nurse leader needs to be able to easily and swiftly adjust to situations around them, not only understanding them, but provide the appropriate direction to staff in ethical situations to achieve the best outcomes [16].

Being able to communicate is important. A leader needs to show a willingness to listen [18]. Through listening and effective communication, nurse leaders can be alerted to many workplaces' ethical problems and put into place action plans, enhancing a healthy working environment [18]. Establishing good communication within the team may promote increased interprofessional collaboration, building an environment where nurses feel empowered to speak up [19].

Leaders who actively assist with daily nursing tasks were described as important in managing distress [20]. Being available and proactive by assisting nurses on the floor with patient care when the need arises was viewed favorably [20]. This presence, from a nurse leader, showed that they are part of, and want to be part of, the team [20].

Nurse leaders (at all levels) need to be able to advocate for the patients, family and fellow nursing staff, and must do so with empathy [19, 21]. Empathy is important as it assists in relating and effectively communicating with patients and staff [21]. The research literature also suggests that the number of years in nursing practice and the quality of this nursing experience also impact on the leadership provided [16, 19, 22–24], as the exposure to situations and events provides them with an understanding of the nursing environment [22]. There are different skills required in exercising a response to a distressing situation. Nurse experience is necessary in a leadership position due to the requirement of understanding goals and responsibilities and recognizing certain situations that need an ethical reaction [16].

A positive moral and ethical attitude, appropriate decision-making, self-discipline, responsibility and being clear about standards through good communication are deemed important leadership qualities [25]. A leader who is confident and has a strong sense of professional values will be an effective leader in nursing and readily able to manage situations of moral distress when they arise [25].

### 4.2. Workload and Resource Management

Part of being a good leader is foreseeing potential issues that may arise, particularly through effective workload and human resource management. Prioritization is required in nursing and many factors such as reduced staffing and increased patients with challenging needs, can all affect time, impacting on the ability to achieve tasks required, which can be a factor leading to moral distress [26]. Prioritization is required by the leader to ensure adequate distribution of resources and management of patients. The literature suggested that there are often tensions around managing workloads and supporting staff [26]. Some leaders tend to prioritize organizational demands instead of their human and ethical ones, leading to a more top-down approach to leadership [26]. This can create significant challenges for nurses attempting to prioritize quality care for patients, or who require support through distressing situations. Intrapersonal skills of rationality and rebalancing practices are important in workload management as they increase collaboration and prevent nurses on the ward being overloaded, as well as promoting integrity and well-being of staff [27].

Time is the biggest consideration with workload management [26]. Not having enough time impacts on nursing workloads and the care they provide to patients [26]. Nursing staff expect that the nurse leader can resolve conflict or a situation in a timely manner [23]. This can be difficult to achieve for the nurse leader, due to competing priorities and directions.

Resource allocation is important with workload management [18]. Equipping the clinical space with the appropriate resources that are used effectively and allocated fairly appears to alleviate some stress in nurses. Furthermore, having the appropriate skill mix, and staffing numbers is important for nurses in managing their workload [19, 24].

Nurse leaders need to have ethical knowledge to effectively manage staff workloads. Having this knowledge is important, as being sensitive to possible ethical situations and implications will ensure that patient and nurse safety is prioritized, and in turn assist with the prevention of moral distress [28].

### 4.3. Culture and Values

Establishing a healthy culture and positive values in the workplace is important, with nurse leaders playing an essential role in driving culture and values. There are many ways a leader can provide support, such as having increased knowledge both clinically and ethically, opening discussions within the team and ensuring adequate resources [26]. Nurse leaders in middle management roles such as clinical nurse specialists, or nurse managers, often find themselves caught between more senior staff, hospital bureaucrats and nurses on the ward [26]. It is crucial that the environment nurses work in is a supportive one, where they feel comfortable speaking up, are part of a team and empowered [27, 29]. A positive culture and workplace values will assist in creating an enjoyable and safe environment for patients and nurses. Nurse leaders need to affirm and validate feelings, and commit to addressing issues and turning negative effects into motivation for improvement, which builds a supportive environment [17, 20, 25]. Nurse leaders themselves need to be resilient in order to establish resilience in others, and they must know how to effectively manage moral distress and compassion fatigue [30]. Compassion fatigue is frequently misinterpreted as burnout and is usually caused by workplace stressors, and this impacts on moral distress [19]. Compassion fatigue is a unique form of burnout, often resulting from a continuous feeling of not being able to give the care that is deemed appropriate, which is very similar to moral injury [31].

Moral distress can arise when there are differences in values [26]. Responding positively when a nurse escalates or advocates for a patient is important, as it demonstrates the leader cares about the nursing faculty and has human caring values [29]. In addition, leaders on the floor are often torn between those they advocate for and those they report to [22]. Culture is impacted heavily by leadership. Leadership sets the scene for driving engagement in the clinical setting [32]. Furthermore, a positive culture from effective leadership can assist in developing resilient nurses who are able to manage problems more effectively. It also encourages stronger teamwork and lower stress levels in the working environment [32].

Ethics is part of culture in a nursing workplace. Ethics in nursing refers to nurses demonstrating values of the profession which include respect, justice, empathy, responsiveness, caring, compassion, trustworthiness and integrity [12]. Leaders employing an ethical leadership style can have a significant impact on the culture of the workplace [17]. Nurse leaders understanding the Code of Ethics is crucial [29]. Of importance is staff involvement in ethical discussions, leading back to effective communication [21, 33]. It is important that leaders conduct ethical decision-making and include the team in this process [34]. A strategy for nurse leaders to help their workplace become more ethical is to foster ethical champions or establish a nursing ethics council [35]. Adopting a more ethical approach, communicating, advocacy, understanding the needs of the ward and engaging in reflection are all part of creating positive values and a healthy culture [24, 26].

## 5. Discussion

While there is significant research literature on leadership in nursing, there is less on the topic of the role of leadership in supporting staff around and mitigating moral distress. Through this scoping review of the literature, three key categories were generated: qualities of a nurse leader, workload and resource management and culture and values.

Given the challenging nature of the profession, nurses look to leaders for support, guidance, decision-making and direction. The qualities that the leader possess are thus essential, as is the ability to navigate complex situations in the workplace. This requires a depth of nursing experience and knowledge. Self-awareness, authenticity, years of experience, effective communication and advocacy for both patients and staff are highlighted qualities that nurse leader must possess. These qualities draw on intrinsic values, but can also be taught and nurtured through mentoring, skills development and higher education [27]. As such, the development of these skills in nurse leaders is an important endeavor for health services.

Likewise, nurse leaders must also have technical leadership skills, such as the ability to manage workload, effectively prioritize resources and staff, in an effort to manage time, to prevent nurse burnout leading to moral distress [27]. The final theme presented was positive culture and values, which drives the need for empowerment, connection to workplace and professional values and the need for leaders to champion this. What is particularly important within the literature was the need for leaders to translate two worlds; the organization enforcing policy, and the staff on the ward doing the work [21, 22, 29]. Leaders may often feel like the ‘middleman' between what can be opposing views of care delivery. While healthcare systems can invest in leadership training and cultural identity, a tangible change that they must enact is system-wide issues including policy and procedure such as workload requirements which support adequate staffing levels, resources and escalation pathways. The need for reform in this area is multifaceted involving barriers to effective nursing leadership, including existing power differentials, inconsistent connectedness with physicians and other healthcare staff, a lack of early socialization experiences with their leader and the need for clinical practice reform which priorities mutual responsibilities [33]. This is of critical importance; social and organizational issues, including power struggles within the system impact upon the delivery of person-centred care [34].

The recent pandemic highlighted fractures in the nursing system and these fractures and complexities can be difficult to navigate for a nurse leader. Beyond the pandemic, nursing is a complex role. Managing the care of vulnerable people within the confines of the health system is challenging, and the impacts of this can be damaging. While the pandemic may have highlighted issues of moral distress in nursing, it only exacerbated what was already there for many nurses [36]. As such, exploring the role of nurse leaders in supporting colleagues through and mitigating some of the effects of moral distress is a timely and important inquiry. A gap in the literature regarding leadership and moral distress exists. While other researchers have explored the impacts of moral distress on nursing leaders themselves [4, 14, 28], this review explored the role of leadership in identifying, mitigating and supporting staff through such challenges. This is an important avenue of inquiry.

To support nurse well-being and quality patient care, supported nursing leadership which mitigates moral distress is essential. Indeed, supporting frontline nurses to think and act ethically within a supportive environment requires systemic change that healthcare leaders must consider. In fact, it is imperative, given that moral distress can, and will, lead to further attrition within the profession.

### 5.1. Implications for Future Research

This review has highlighted the importance of leadership in identifying and mitigating the impacts of moral distress. This is important, as future research, training and leadership processes must place a focus on the role of the leader in supporting staff through moral distress, a phenomenon which is increasing in frequency and consequence. In relation to policy change, the implementation of moral distress support services requires further attention and research.

### 5.2. Limitations

This scoping review was limited to English language articles, and as such, knowledge from other sources may have been missed. Similarly, only peer-reviewed literature written in English was included; therefore, gray literature was not included. Both limitations may have contributed to potential publication bias. However, by following the processes of the scoping review, and detailing strict parameters for publication selection, the review offers robust insights into the available published literature.

This review focused on the leadership of nurses and moral distress, and lessons from other professions may be valuable, particularly regarding how leaders can support and potentially mitigate it.

## 6. Conclusion

This scoping review explored literature related to the role of nurse leaders in supporting and mitigating moral distress. Three themes were identified as crucial elements to improving moral distress. These are the qualities of the leader, managing workload and resources, and fostering a healthy culture with positive values. Retaining a suitably qualified nursing workforce is essential to addressing gaps in health service delivery as well as the current and predicted nursing workforce shortages; therefore, effective nurse leadership can assist with this current issue and the prevention of moral distress. Organizational, multidisciplinary efforts are required to support nurse leaders to care for staff experiencing moral distress; this is a fruitful area for future research to focus on.

## Figures and Tables

**Table 1:** Search terms.

PICO	Search terms
Population	‘Registered Nurse', Nurse, Nurses, Nursing, ‘Nurse leaders', ‘Nurse supervisors', ‘nurse administrators' AND
Intervention	Leadership, Leader, Leaders AND
Context	‘Moral Distress', Distress, ‘Psychological Distress', Wellbeing, Well Being, Well-being

**Table 2:** Inclusion and exclusion criteria.

Inclusion	Exclusion
Participants—Registered nurses, nurse managers, nurse leaders	Enrolled nurses, assistants in nursing
Concept—Leadership, management, moral distress	Moral injury, post-traumatic stress syndrome (PTSD), potentially morally injurious events, COVID-19 pandemic
Context—Moral distress in leaders, moral distress in nurses and morally distressing situations or events. Only data related to moral distress extracted	Data relating to other specific phenomena (such as psychological distress) not included
Peer-reviewed papers	Non–peer-reviewed papers, editorials, media, thesis, rapid, systematic, integrative and scoping reviews
Empirical articles published between 2022 and 2025	Papers published before 2022.
Full text, English language	Non-English publications.

**Table 3:** Categories and subcategories within the literature.

Category	Subcategory
Qualities of the leader [15–25]	Self-awareness
	Authenticity
	Experience
	Communication
	Advocacy

Workload and resource management [18, 19, 23, 24, 26–28]	Effective prioritization
	Time
	Resource management

Culture and values [12, 17, 19–22, 24–27, 29–35]	Being supporting
	Driving a culture of safety
	Encouraging nurses to advocate and work to values

**Table 4:** Medline (Ovid).

Search	Query	Records retrieved
#1	Registered Nurse.mp.	4657
#2	Exp nurses/	101,579
#3	Nurse.mp.	204,778
#4	Nurse leaders.mp.	2632
#5	Nurses.mp.	288,799
#6	Nurse supervisor.mp.	171
#7	Exp nursing, supervisory/	8433
#8	Exp nurse administrators/	14,336
#9	Nurse administrators.mp.	172
#10	1 or 2 or 3 or 4 or 5 or 6 or 7 or 8 or 9	415,010
#11	Exp leadership/	49,590
#12	Leadership.mp.	83,621
#13	Leader.mp.	24,496
#14	Leaders.mp.	45,834
#15	11 or 12 or 13 or 14	132,855
#16	Moral distress.mp.	2186
#17	Distress.mp.	186,744
#18	Exp psychological distress/	8017
#19	Psychological distress.mp.	33,601
#20	Wellbeing.mp.	35,761
#21	Well Being.mp.	134,862
#22	Exp Australia/	178,775
#23	Australia.mp.	208,038
#24	16 or 17 or 18 or 19 or 20 or 21 or 22 or 23	564,478
#25	10 and 15 and 24	1537
#26	Limit 25 to English language	1524
#27	26 and 2022:2025 (sa_year).	528

*Note:* Search conducted 24 October 2024.

**Table 5:** Extraction tool.

#	Citation	Findings	Moral distress definition	Themes of moral distress in nursing	Leadership qualities regarding moral distress and the impact of this	How do nurse leaders manage moral distress
1	Abousoliman, A., D., Mahmoud. et al. (2024). Effect of authentic leadership on nurses' psychological distress and turnover intention. International Journal of African Nursing Sciences [37].	Authentic leadership style had a statistically significant negative relationship with psychological distress (*r* = −0.28), and turnover intention (*r* = −0.18). There is a statistically significant positive relationship between psychological distress and turnover intention (*r* = 0.52).	A response to stressors such as depression, anxiety, burnout, that is characterized by a sequence of poor psychological cognitions, emotions, behaviours, and other psychological manifestations.	• Depression, Anxiety, burnout • Turnover • Decreased nursing morale	• Authentic leadership • Self-awareness • Internalized moral perspectives • Balanced processing • Relational transparency	• Leaders who are true to themselves demonstrate authenticity in their interactions with staff • These actions are important for improving employee perceptions of support and reducing employee turnover • It is imperative for nurses and healthcare systems to have knowledge of psychological distress risk factors and turnover intention predictors.

2	Ahokas, F., Hemberg, J. (2023). Moral distress experienced by care leaders' in older adult care: A qualitative study [26]	Five themes: 1. Moral distress arises from a lack of time 2. Moral distress contributes to a sense of inadequacy but also a sense of responsibility 3. Moral distress arises from an imbalance in values, 4. Increased knowledge and open discussion help reduce moral distress and 5. Reflection, increased support and increased resources can reduce moral distress	Defined as a psychological imbalance that occurs when people are aware of the ethically appropriate action that a situation would require but cannot perform this action due to external obstacles	• Lack of time • Contributes to a sense of inadequacy but also a sense of responsibility • Arises from an imbalance of values	• Discussing moral distress outside of the workplace was not possible because of their duty of confidentiality and noted that work super-vision was possible, perceiving speaking to an outside expert or therapist to be an important support function • Increased knowledge and open discussions help reduce moral distress	• Reflection, increased support and increased resources can reduce moral distress.

3	Albaqawi, H. M., Alshammari, M. H. (2024). Resilience, compassion fatigue, moral distress and moral injury of nurses [30].	Compassion fatigue had a direct, positive effect on moral distress and moral injury, and moral distress had a direct, positive effect on moral injury.	Moral distress occurs when nurses encounter situations in which they are compelled to act ethically but are unable to do so due to internal or external factors beyond their control.	• Resilience had a positive impact upon moral distress. • Nurse leaders may play a significant role in fostering nurse resilience and promoting a sustainable nurse workforce by supporting professional training and social support	• Leader should adopt and strategies and initiatives that promote resilience and effective management of compassion fatigue, moral distress, and mental illness.	• Support resilience and reduce compassion fatigue • Leader must recognize individuals, yet the shared experience of compassion fatigue

4	Al-Rjoub, S. F., Diener, E. et al. (2022). Nurse administrators as the cause of moral distress among nurse educators: A qualitative research study [29].	Five themes a. Administrative support deficit b. Administrator-faculty member rapport c. Sense of powerlessness d. Marginalization in the decision-making process, and e. Being forced to work in opposition to the nursing profession value system.	Knowing the moral actions to take but being constrained from such actions. Moral distress can lead to psychological, emotional, and physical symptoms among nurses such as anger, frustration, guilt, withdrawal, depression, gastric upset, headaches, and palpitations	• Lack of administration support leads to being overwhelmed • Feeling of powerless can be overcome by involving nurses in the decision-making process	• Leaders play a vital role in preventing moral distress by providing management that demonstrates caring human values and advocates for nursing faculty • Leader should create a balance between the organizational requirements and the needs of the nursing faculty members.	• Being aware of code of ethics • Create an environment that supports nurses to speak up and empower them • Ensure resources are available to leaders and managers to assist those in overcoming moral distress

5	Ashida, K., Kawashima, T., et al. (2022). Moral distress among critical care nurses: A cross-cultural comparison [22]	Highest moral distress scores were those related to aggressive/inappropriate treatment. The total MMD-HP score was significantly higher in Japanese nurses compared to US nurses (122.8 ± 70.8 vs. 112.3 ± 73.2). Some factors, such as leadership experience, were associated with higher moral distress.	Knowing the right thing to do but being in a situation in which it is nearly impossible to do it.	• Intention to leave the job	• Leadership experience • Nursing experience • Training • Frequency of discussing ethical issues with others	• Nurses may be exposed to organizational rules and regulations, which may make it complicated to act between patient rights and organizational policy.

6	Beil-Hildebrand, M. B., Kundt Sari, F. et al. (2024). What keeps you up at night? Moral distress in nurse leaders in the USA, Germany, Austria and Switzerland [34]	Leader experience moral distress through a lack of individual and organizational integrity, hierarchical and interprofessional issues, lack of nursing professionalism, patient care/patient safety concerns, finances negatively impacting care and issues around social justice.	Moral distress occurs when a moral judgment has been made, and there are institutional constraints that prevent that moral judgment from being acted on.	• Town over • Burnout • Work disengagement • Compassion fatigue • Intention to leave • Frustration, anger, sadness, exhaustion and depression, sleeplessness, nausea, migraines, GI upsets	• Nurse leaders experience moral distress as they are caught in the middle of practice and policy	• Moral reasoning and ethical decision making

7	Clark, P., Hulse, B., et al. (2022). Resilience, moral distress, and job satisfaction driving engagement in emergency department nurses: A qualitative analysis [32]	Participants indicated that greater nursing experience increased confidence in skills, ability to over-come emotional stressors, and more satisfaction with patient care all improved resilience and workplace engagement. Morally distressed, disengaged nurses lacked workplace autonomy and ability to make workplace changes or worked in hostile and unsafe workplaces. Engaged nurses invested more time in their job and were more willing to remain in their workplace.	Knowing and being unable to take the correct course of action owing to 1 or more root causes (patient, unit, system), feeling complicit in providing care that violates core nursing (vs. personal) values, and having no voice to change the course of action.	• Disengagement from work • Low work engagement	• Leadership played a role in helping or hindering resilience in terms of job satisfaction and engagement that could impact retention	• Providing empowering and supportive environment nurses then feel resilient and more equipped in managing problems more effectively. When feeling supported nurses were more willing to free up more energy for other work, such as agreeing to additional shifts, committee membership, or social outings with work colleagues. • In providing a culture promoting resilience, ED nurses also reported stronger feelings of staff teamwork. If the team are able to trust and feel they are supported working alongside their co-workers, this is also providing support in an environment of high stress.

8	Faraco, M. M., Gelbcke, F. L., et al. (2022). Moral distress and moral resilience of nurse managers [27]	Personal adaptive strategies (intrapersonal and interpersonal) and organizational collaborative strategies (intrinsic and transformational management) emerged from this process. Involved elements of rationality, flexibility, rebalancing practices, moral courage, and detachment. Organizational strategies dealt with actions which reorient ethical infrastructure, ethical education, and psychological protection, as well as fostering dialogical relationships, empowerment.	Identifying a morally correct decision, however, feel prevented from acting in accordance with their conscience and values, due to different types of barriers	• Burnout • Lack of engagement • Little resilience	• Courage • Flexibility • Rationality • Rebalancing practices • Distancing • Dialog • Involving the team • Support network	• Personal adaptive strategies (intrapersonal and interpersonal) and organizational collaborative strategies (intrinsic and transformational management) emerged from this process.

9	Jansen, T. L., Hem, M. H. et al. (2022). Coping with moral distress on acute psychiatric wards: A qualitative study [33]	Various coping strategies were used: mentally sorting through their ethical dilemmas or bringing them to the leadership Not ‘bringing problems home' after work or loyally doing as told and trying to make oneself immune. Colleagues and work climate were important for choice of coping strategies.	Unpleasant feeling or a psychological imbalance which arises when one knows what the ethically right action is, but internal and/or external factors make it impossible to act accordingly.	• Moral distress is an organizational problem experienced at a personal level. • Feelings of guilt, bad conscience, sadness, powerlessness, emotional numbness, shame, cynicism, despondency, anger, angst, self-criticism and resignation.	• Being supportive and empathetic • Debriefing • Appraising staff on efforts • Building an empowering team • Engagement with team • Inclusive • Being attuned to emotional and not coping signals	• These nurses' experiences indicate that staff involvement in ethical discussions should be supported and promoted by the leadership.

10	Johnstone, R., Edwards, P. (2023). Supporting nurse leaders to recognize and mitigate the effects of moral injury [17]	Nurse leaders have an important role in providing supportive interventions before, during and after an event involving potential moral injury. The framework described in this article provides a starting point for nurse leaders attempting to mitigate moral injury	Psychological conflict experienced by nurses during ethical dilemmas. Moral injury occurs when a person perpetrates, fails to prevent, bears witness to or learns about acts that transgress their deeply held moral beliefs or expectations	• Nurses are morally sensitive to the vulnerability of patients in their care. • Nurses experience organizational or systemic factors preventing them from doing what they feel is best for patients. • Nurses feel that they cannot influence or control the specific situation	• Confidence • Increase knowledge and understanding of moral distress • Applying an ethical leadership style. • Resilience	• Affirm and validate feelings by openly talking to others and making a commitment to address the issue. • Assess the underlying causes of moral distress. • Act to address the specific causes, turning the negative effects into motivation for improvement. • Early intervention

11	Kok, N., Van Gurp, J. et al. (2023) complex interplay between moral distress and other risk factors of burnout in ICU professionals: Findings from a cross-sectional survey study [20]	Moral distress was directly associated with emotional Exhaustion (*β* = 0.19, *p* < 0.05). The association between moral distress and emotional exhaustion was also mediated through negative work-to-home Spillovers (*β* = 0.09).	A psychological response to morally challenging situations such as those of moral constraint or moral conflict, or both.	• Emotional exhaustion and depersonalization. • Carries over into work-home, private life exacerbating symptoms leading to state of physical and emotional exhaustion	• Leadership support • Leaders attuned to emotional signals • Willingness to listen and to participate on an equal footing.	• Support from supervisors moderates the association between moral distress and emotional exhaustion • To share their concerns and the opportunity to exert some influence over moral decisions, and • Consistently reprimand unacceptable behaviour of professionals towards each other.

12	Maximiano Faraco, M., et al. (2022). Moral distress-associated sociodemographic and occupational aspects in nursing managers at federal university hospitals [16].	The highest levels of moral distress were experienced by nurses in large hospitals, with statistical significance among civil servants with job stability who have no management training, with less time of professional experience and with the highest weekly workload.	Physical and emotional manifestations, arising from a process of recognition and perception of morally conflicting situations, and the awareness of the morally correct action that, due to institutional or social impediments, is not carried out, threatening fundamental Values and moral integrity	• MD higher is in younger nurse managers • No differences between genders • Higher rates of MD in the larger hospitals • Higher number of years since graduation lower rates of MD, higher number of years as manager lower rates of MD • Hours of work impact on MD- higher hours have higher rates. • Nurses with training in management have lower rates • Nurses with firm and permanent contracts have higher rates of MD	• Complementary training, training in the management area • Time of work as nurse, time of experience in management • Time of experience in the function, number of bonds, type of bond, role, and weekly workload.	• Manager learns and equips themselves with skills to manage MD and ethical situations • Nurse leader being ethically sensitive • Effective communication • Provide a safe, supportive and empowering working environment

13	Miller, P. H., Epstein, E. G. et al. (2024). Critical care nurse leaders addressing moral distress: A qualitative study [21]	ICU nurse leaders can recognize and resolve MD in their team. Nurse leaders can experience MD themselves and the high stress role can exacerbate their MD.	Occurring when nurses or other clinicians are constrained from taking what they believe to be ethically appropriate actions or are forced to enact in an ethically inappropriate manner based on their professional obligations, resulting in a sense of complicity and wrongdoing	• Unavoidable for nurse leaders • Feeling powerless precipitates MD • Difficult end of life situation • System barriers, eg policies inconsistent with values	• Accessing internal organizational resources and support • Seeking external resources • Access to Employee assistance programs. These programs were described as offering therapy and counselling	• Prioritize, budget for, and monitor outcomes related to well-being initiatives • Refine policies and processes and shape the work environment and culture to promote access to adequate resources • Provide support for individual nurses in navigating work-system stressors. • Implementation of a system-wide moral distress consultation service

14	Schulz, I., O'Neill, J. et al. (2023). The scope of ethical dilemmas in paediatric nursing: a Survey of nurses from a tertiary paediatric centre in Australia [35]	Knowledge and use of the clinical ethics service was poor and the most frequent challenge for nurses in managing dilemmas was feeling powerless.	Does not define moral distress concisely, gives examples of what can cause moral distress	• Exposure • Directive from leadership to be unethical • Complex situations providing challenges • Increasing acuity • Nursing area	• Leaders and managers in the paediatric setting need to understand the type and range of ethical issues facing bedside nurses in order to ensure there are adequate resources and space for ethical reflection, and so that they can respond to ethical issues in a proactive manner	• Fostering ethics champions to empower nurses within ward areas to act as clinical ethics resources • Recognition of the different profile of ethical dilemmas in the paediatric critical care setting has been managed by innovations such as formal weekly discussions of patients with prolonged lengths of hospitalization • Establishment of a nursing ethics council • New strategies for ethics education and ethics support need to be led and perhaps provided by nursing leadership and management, in collaboration with the clinical ethics service

15	Semler, L. R. (2023). Moral distress to moral success: Strategies to decrease moral distress [18]	Nurses experienced a significant decrease in moral distress. The participants' average ethical confidence increased in four areas (ability to identify the conflicting values at stake, knowing role expectations, feeling prepared to resolved ethical conflict, and being able to do the right thing)	Knowing the correct action to take, but being unable to pursue that action due to internal or external constraints	• Communication with team • Disproportionate suffering of patients/families • Unclear goals of care • Patients' decision-making capacity • Resource allocation	• Nurses have a higher incidence of moral distress than physicians, likely due to the nurse's perceived inability to make decisions and their feeling of being “voiceless” during morally complex conversation • It is especially important for clinical leaders to address moral distress in their nursing staff	• Education • Ethics huddles and discussion • Group discussion and/or debriefing • Self-reflection • Identifying barriers • Creating personal action plans • Communication strategies.

16	Sheppard, K., Smith, C. et al. (2024). The effect of nursing moral distress on intent to leave Employment [19]	MD was significantly higher among RNs intending to leave employment. System-level and team-level integrity attributes were significant factors predicting intent to leave, controlling for potential confounders.	Describes the lived experiences one faces when actions and beliefs contradict, or beliefs of what is right or wrong counteract desired outcomes	• Newly graduated and 2^nd^ RNS mostly affected • Managing patient care, within organizational constraints, may exacerbate MD and negatively impact RN retention • Ethics and work environment factors • Staffing shortages • Perceived lack of organizational support regarding patient care issues • Power hierarchies within the work environment • Poor communication with leaders • Perceived lack of education when caring for complex patients	• Being supportive • Integrity • Understand types of ethical issues and their impacts.	• Identify and intervene to promote a healthy work environment • Interprofessional collaboration, skilled communication, decision latitude, appropriate staffing, meaningful recognition, and authentic leadership • Improving the workplace environment

17	Stanojević, S., Čartolovni, A. et al. (Date). Moral distress and moral injury and their interplay as a challenge for leadership and management: The case of Croatia [23]	Strong correlation between moral distress and moral injury, but also in terms of nurses' decision to leave or consider leaving their position.	Moral distress by way of external and internal constraints is a situational problem relatively easy to prevent	• High stress in position • The decision made not complying with ethical standards • Weakness in effective communication	• Support and supervision from leaders • Situational awareness • Years of experience in position • Years of education • Work-break • Cause of work break	• The awareness and recognition of moral injury still need to transpire, as some leaders might also suffer from it • Timely recognition and distinction between moral distress and moral injury may assist nursing managers and leaders in acting and responding timely

18	Tavakol, N., Molazem, Z. et al. (2022). Moral distress in Iranian psychiatric nurses: A content analysis [24]	Safe work environment to be established by managers by means of appropriate staffing and needs of nurses	Moral distress occurs when a person knows what is morally right but is prevented from doing it due to organizational barriers	• Weaknesses in professional and effective communication	• Lack of professional competence • Organizational culture • Individual, organizational and environmental factors • Management factors • Observation of moral dilemmas by nurses • Understanding of needs of the ward and nurses • Appropriate supervision required	• Providing a safe environment and appropriate facilities for psychiatric nurses • Provide sufficient nursing staff to reduce the workload of psychiatric nurses and provide the necessary facilities for psychiatric hospitals • Creative methods of staffing management (such as organizing the proper division of work and applying adequate supervision) and reducing the workload of nurses.

19	Waterfield, D., Barnason, S. et al. (2024). It kills your soul: A Mixed methods study of ethical sensitivity of critical care nurses [28]	Themes of ambiguous beneficence, heedless autonomy, and moral distress were found to be related to pain, agitation/sedation, delirium, immobility, and sleep disturbances	Not provided	• Ambiguous Beneficence—inconsistency • Communicating with the patient—accuracy • Comforting the patient—sedation • Heedless autonomy—struggles with incongruence • Conflicting with hierarchy—lack of knowledge of nurse-driven interventions & misinterpreting symptoms • Moral distress—deprioritizing pain, omitting best practice	• Ethical knowledge • Positive moral attitude • Appropriate decision making • Self-discipline • Responsible • Clear about standards	• Visibility • Clear direction • Establishing supportive work environment

20	Yu, Q., Wang, H. et al. (2023). Moral courage, job-esteem, and social responsibility in disaster relief nurses [25]	Disaster relief nurses' moral courage positively impacted social responsibility, and moral courage could affect social responsibility through the mediating role of job-esteem.	Not provided	• Low clinical experience • Younger nurses • No leadership training opportunity • Confidence level • Marital status—not being well supported • Lack of courage to defend patients • Speaking up may mean they are being retaliated or ostracized.	• Longer work experience • Strong sense of professional value	• Leadership training • Nursing managers regular assessment of nurses' moral courage and interventions such as meetings and

## Data Availability

The data that support the findings of this study are available from the corresponding author upon reasonable request.
